# Detection of Anti-*Toxoplasma gondii* IgG and IgM Antibodies and Associated Risk Factors during Pregnancy in Southwest Iran

**DOI:** 10.1155/2021/5547667

**Published:** 2021-05-27

**Authors:** Shahrzad Soltani, Ali Dalir Ghaffari, Mehdi Sagha Kahvaz, Mohamad Sabaghan, Marzieh Pashmforosh, Masoud Foroutan

**Affiliations:** ^1^USERN Office, Abadan University of Medical Sciences, Abadan, Iran; ^2^Department of Parasitology, Faculty of Medical Sciences, Tarbiat Modares University, Tehran, Iran; ^3^Behbahan Faculty of Medical Sciences, Behbahan, Iran

## Abstract

**Background:**

This research was aimed at evaluating the seroprevalence of acute and chronic *Toxoplasma gondii* (*T. gondii*) infection in pregnant women and related risk factors in southwest Iran.

**Methods:**

In this cross-sectional study, eighty-eight pregnant women were included from October 2019 to December 2019. The presence of anti-*T. gondii* IgM and IgG antibodies was measured using the enzyme-linked immunosorbent assay (ELISA). In addition, a questionnaire consisting of demographic information was completed for each subject.

**Results:**

The overall seroprevalence of *T. gondii* infection was estimated to be 34.09% (30/88). Of these, 1 (1.13%) and 29 (32.95%) samples were found positive for IgM and IgG, respectively. Regarding the risk factors, the consumption of raw/undercooked meat (*P* value = 0.007) and history of abortion (*P* value = 0.017) were significantly associated with IgG seroprevalence in pregnant women.

**Conclusion:**

The results showed that the pregnant women of southwest Iran might be moderately exposed to *T. gondii*. Since the risk of acute *T. gondii* infection in this susceptible group is very important, regular screening tests to diagnose the infection are recommended before pregnancy.

## 1. Introduction

*Toxoplasma gondii* (*T. gondii*), an obligate intracellular parasite from the phylum Apicomplexa, is a zoonotic protozoan that infects numerous warm-blooded vertebrate species like humans, livestock, birds, and aquatic mammals [1, 2]. This parasite impresses one-third of the global population, especially in low-income and developing countries [3, 4]. Overall, *T. gondii* requires two hosts to complete its environmental life cycle. Cats (family: Felidae) are the final hosts, whereas most warm-blooded animals act as intermediate hosts [1]. The parasite is transmitted mainly through the following routes: intake of raw/undercooked meat infected with tissue cysts, drinking the water or consumption of unwashed vegetables infected with oocysts, and infection from mother to fetus [5].

The high-risk groups for *T. gondii* infection are immunocompromised individuals such as patients with cancer, HIV-positive people, and organ transplant recipients [4]. Moreover, *T. gondii* in those seronegative pregnant women who acquire the infection during their pregnancy period may cause serious abnormalities in the fetus [6, 7]. Pregnant women generally show no signs of this infection throughout pregnancy. The fetus is likely to be subjected to congenital infection upon maternal infection. The intensity of vertical transmission and harm to the embryo depends entirely on the date of the infection occurring during pregnancy. The longer the time elapses throughout pregnancy, the more parasites can invade the fetus. The disorders after congenital transmission include focal necrosis, inflammation, and disorders like brain and eye injuries. In the case of severe infection, there may be other consequences including microcephaly, hydrocephalus, mental retardation, and deafness [8, 9]. In the absence of prenatal screening programs in a specific region, the high percentage of acute *T. gondii* infections in this susceptible group will remain overlooked and left untreated [9, 10]. To this end, the objective of this research was to evaluate the seroprevalence of *T. gondii* infection in pregnant women and their related risk factors in southwest Iran.

## 2. Materials and Methods

### 2.1. Study Area

Abadan City is located in the southwest of Iran. This city is bordered to the west by the Arvand waterway and to the east of the Karun River (Arvand Rood) Bahmanshir outlet near the Iran-Iraq border. Now, its population reaches nearly 350,000 people. Summers are dry and intensely hot, with temperature up to 53°C. Abadan is particularly one of the few warmest crowded places in the world with several storms of sand and dust annually [11].

### 2.2. Study Population

In this cross-sectional survey, the study population was pregnant women who were referred to the Taleghani and Beheshti hospitals affiliated to the Abadan Faculty of Medical Sciences from October 2019 to December 2019 in the southwest Iran (Abadan County, Khuzestan Province). Accordingly, eighty-eight blood samples were collected from pregnant women. All pregnant women voluntarily agreed to be examined. A written informed consent form was completed and signed by each subject. The study protocol was approved by the Ethical Committee of the Behbahan Faculty of Medical Sciences (IR.BHN.REC.1399.007).

### 2.3. Questionnaire

A questionnaire consisting of demographic information was completed for each person, including age (10-20, 21-30, 31-40, and 41-50), place of residence (urban or rural), education level (diploma or lower and university degree), reason for referral (routine checkup or abortion), contact with cat (yes or no), history of abortion (yes or no), consumption of raw/undercooked meat (yes or no), and source of drinking water (purified or unpurified water).

### 2.4. Serology

Five milliliters of women's blood samples was taken. All the blood samples were transferred to the central laboratory of the Abadan Faculty of Medical Sciences. First, the blood samples were centrifuged for five minutes at 4000 rpm. The sera were then separated and placed at -20°C until analysis. The presence of anti-*Toxoplasma* IgM and IgG antibodies was evaluated via the enzyme-linked immunosorbent assay (ELISA), with an ELISA kit (Torch-IgG, IgM-Trinity Biotech Company) according to the manufacturer's instructions. The samples with international units (IU)/ml of <0.9, 0.9-1.1, and >1.1 were considered negative, borderline, and positive, respectively [11].

### 2.5. Statistical Analysis

All data were imported into the Statistical Package for the Social Sciences (SPSS) software (version 21) (SPSS Inc., Chicago, IL, USA) for more analysis. For this purpose, the Pearson chi-square and Fisher exact tests were used. The significance level was defined to be less than 0.05.

## 3. Results

### 3.1. Seroprevalence of Anti-*Toxoplasma* Antibodies

In this research, 88 pregnant women were screened by the ELISA method for the presence of anti-*Toxoplasma* IgG and IgM antibodies. The overall seroprevalence of *T. gondii* infection was 34.09% (30/88). Of these, 1 (1.13%) and 29 (32.95%) samples were identified positive for IgM and IgG, respectively (Table 1).

### 3.2. Risk Factors

Eight risk factors related to *T. gondii* infection were recorded in this study. In terms of the residence, 30.35% (17/56) and 40.62% (13/32) of the subjects from urban and rural communities were seropositive for *T. gondii*, respectively (*P* value = 0.493 and 0.364 for IgG and IgM, respectively). All the participants were divided into four categories which include 10-20, 21-30, 31-40, and 41-50 years, and the exposure to *T. gondii* was estimated as 20% (3/15), 38.09% (16/42), 34.61% (9/26), and 40% (2/5), respectively (*P* value = 0.711 and 0.775 for IgG and IgM, respectively). In addition, a higher seropositivity was found in participants with a diploma or lower education level (38.98%; 23/59) (*P* value = 0.217 and 0.481 for IgG and IgM, respectively), in individuals who had direct contact with cats (41.02%; 16/39) (*P* value = 0.327 and 0.443 for IgG and IgM, respectively), in individuals who drank unpurified water (52.17%; 12/23) (*P* value = 0.077 and 0.75 for IgG and IgM, respectively), and in individuals who were referred due to abortion (50%; 5/10) (*P* value = 0.724 and 0.898 for IgG and IgM, respectively). In the above-mentioned risk factors, there was no significant association between the seroprevalence of *T. gondii* infection and IgG and IgM antibodies, but there was a statistical association between the IgG level and history of abortion (60%; 12/20) (*P* value = 0.017 and 0.277 for IgG and IgM, respectively) and consumption of raw/undercooked meat (52.94%; 18/34) (*P* value = 0.007 and 0.386 for IgG and IgM, respectively). Table 1 summarizes the main features and risk factors associated with *T. gondii* prevalence rates in southwest Iran.

## 4. Discussion

Acute *T. gondii* infection in pregnant women may trigger congenital toxoplasmosis if ignored. The infection can lead to significant and progressive diseases with high morbidity and mortality in fetuses and newborns [8, 9]. Therefore, in this study, we evaluated the prevalence of specific *T. gondii* IgG and IgM in pregnant women in Abadan City, southwest Iran.

Numerous studies investigated the prevalence of *T. gondii* infection in pregnant women, which differs across regions. For example, findings in the Americas and Africa have shown a higher prevalence, ranging from 6.1% to 77.5% and 25.3% to 75.2%, respectively. Also, in Europe and Asia, the seroprevalence rate is between 9.1–63.2% and 0.8–60.4%, respectively [12]. As shown in a recent review article with a meta-analysis approach by Torgerson and Mastroiacovo, the annual incidence of congenital toxoplasmosis has been estimated to be 190,100 cases (179,300-206,300) worldwide [10]. In this survey, 88 pregnant women were tested for the presence of anti-*Toxoplasma* IgG and IgM antibodies. The overall seroprevalence of *T. gondii* infection was 34.09% (30/88) in Abadan City. In two different global meta-analysis studies, the seroprevalence of latent and acute *T. gondii* infection in pregnant women was estimated to be 33.8% (95% CI: 31.8-35.9%) and 1.1% (95% CI: 0.9–1.2%), respectively [3, 9]. In another report by Foroutan-Rad et al., the pooled seroprevalence of *T. gondii* infection among the Iranian pregnant women was 41% (95%CI = 36–45%) [13]. Thus, the seroprevalence rate of the current study was comparable with the Iranian pregnant women's average seroprevalence. In a study by Akhlaghi et al. performed on 400 pregnant women in Karaj, the presence of anti-*Toxoplasma* IgG and IgM antibodies was 29% and 1%, respectively [14]. In Khorramabad [15], the seroprevalence rate of *T. gondii* infection in pregnant women was estimated to be 31%, which is in accordance with the current study, whereas the seroprevalence rate in pregnant women in Kermanshah [16], Isfahan [17], and Sabzevar [18] was 22.7%, 20.1%, and 19.2%, respectively, which is lower than the results of the current study.

There are several probable risk factors for acquiring the *T. gondii* infection, although there are abundant inconsistent outcomes. In this study, a questionnaire was used to document eight relevant risk factors associated with *T. gondii* infection in pregnant women. Upon analysis, we found a statistically significant association between IgG seroprevalence and raw/undercooked meat intake (*P* value = 0.007) and history of abortion (*P* value = 0.017). The other risk factors showed no significant association. Intake of raw/undercooked meat is often seen as a major risk factor for the infection [19]. Consumption of raw or half-cooked meat has been reported as one of the most significant sources of human infection [20]. According to Jones et al.'s study, working with meat and consuming undercooked meat are two of the most significant risk factors for *T. gondii* infection in the United States [21]. The findings of the current study demonstrated a meaningful association between the consumption of raw or half-cooked meat and *T. gondii* infection (*P* value = 0.007). In line with our study, some studies believed that eating raw/undercooked meat could significantly increase the infection rates in societies [22, 23]. For example, in a study by Khademi et al. in Hormozgan Province (southern Iran), a significant association was observed between the consumption of raw/semi-raw meat and *T. gondii* infection [24]. In contrast to our research, there was no statistically different between the consumption of raw/semi-raw meat and toxoplasmosis in surveys conducted by Ertug et al. [25] and Eshratkhah et al. [20]. A study by Rasti et al. in 2017 showed that the prevalence of *T. gondii* infection in sheep and goats ranges from 3.3 to 38% in various parts of Iran. In addition, they showed that sheep and goat are important reservoirs of *T. gondii* [26]. Therefore, the researchers revealed the role of meat and other meat products as sources for *T. gondii*.

In this study, a significant association between *T. gondii* and the history of abortion was observed (*P* value = 0.017). Similar to our study, in studies by Hosseinzadeh et al. [27], Aali et al. [28], and Sahwi et al. [29], the seroprevalence of *T. gondii* IgG and IgM in pregnant women with a history of abortion was statistically higher than that in control groups, which indicates a potential association between *T. gondii* infection and the abortion. However, in contrast with our study, some studies such as Akhlaghi et al. [14], Eshratkhah et al. [20], Babaie et al. [30], and Hajsoleimani et al. [31] did not show any meaningful association between toxoplasmosis and history of abortion. Also, in a study by Ghasemi et al. which evaluated the role of toxoplasmosis in etiology of abortion and stillbirth, the results showed that the IgG seroprevalence was 25.5% in the case group (26.8% in abortion and 21.4% in stillbirth), and no statistically significant differences were seen in the case and control groups [32].

To assess *T. gondii* seroprevalence among various age groups, the participants were categorized into four groups, including 10-20, 21-30, 31-40, and 41-50 years. The seroprevalence rate was 20%, 38.09%, 34.61%, and 40% in these groups, respectively. Although there was no significant association between *T. gondii* infection and different age groups in the current study (*P* = 0.711 and 0.775 for IgG and IgM, respectively), the seroprevalence rate in 41-50 years was higher than that in other groups, which is in accordance with Alvarado-Esquivel et al. [33], Fallah et al. [34], and Daryani and Sagha [35] studies. The seroprevalence rate tended to increase with age. The cause for this may be the rising risk of exposure with age. In a meta-analysis study by Borna et al., in line with our study, the seroprevalence of *T. gondii* IgG increased with age [36].

In the current research, *T. gondii* infection in women who had a history of contact with cats was higher, but the findings were not significant. Nonetheless, a previously published study among the general Iranian population (*P* < 0.05) suggested that contact with cat significantly correlates with *T. gondii* infection [22]. Cats are the only definitive host for the parasites, and due to their close contact with people, especially in the rural areas, there is a general opinion that contact with the cat could be a major risk factor for acquiring the infection [1, 37].

In the current study, 30.35% and 40.62% of pregnant women from urban and rural regions were seropositive for *T. gondii* infection, respectively. Villagers' lifestyles such as direct contact with soil, livestock, and animals could make this situation predictable, as previously reported in most of the published articles [13, 38]. Risk factor assessment in the general Iranian population has shown that *T. gondii* infection can be reduced by growing schooling grades (*P* < 0.0001) [22]. In this study, the same association was observed, but no significant association was observed between *T. gondii* infection and education level. As it is evident, lack of understanding of the primary transmission pathways and sources of infection as well as sanitary conditions are remarkably significant factors for *T. gondii* infection.

This research estimated that the seroprevalence of acute *T. gondii* infection in pregnant women was 1.13% (1/88). In Iran, the seroprevalence of acute infection during the pregnancy was reported to be 1.4% in Zanjan [31], 4.8% in Isfahan [39], 0.5% in Urmia [20], and 0.6% in Kashan [40]. The reason for different infection rates in various areas of Iran and the world may be related to socio-cultural conditions, contact with cats, and observance of health points [41].

## 5. Limitations

Lack of supporting serological data by molecular confirmation and the low number of sample size are the limitations of the study.

## 6. Conclusions

In conclusion, our study revealed an overall seroprevalence of 34.09% with *T. gondii* infection in pregnant women in Abadan City (southwest of Iran). Because the risk of acute *T. gondii* infection in this susceptible group is very important, enhanced control and prevention attempts should be rigorously carried out. In addition, regular screening tests for *T. gondii* infection could also be useful in the routine clinical care of pregnant women. Eventually, improving women's awareness regarding toxoplasmosis and its consequences, the main transmission routes, and associated risk factors can surely decrease the rate of seroprevalence, particularly in rural communities. Health officials should regard the screening test for *T. gondii* infection before marriage or pregnancy as a routine test.

## Figures and Tables

**Table 1:** Demographic characteristics and risk factors related to IgG (latent) and IgM (acute) seroprevalence of *T. gondii* in pregnant women in Abadan County, Khuzestan Province, Iran, from Oct. to Dec. 2019. *P* value < 0.05 indicates a significant difference in seroprevalence between the categories within each characteristic.

Characteristic	Number tested (percent of total tested)	IgG positive	*P* value	IgM positive	*P* value	Total
		Number (percent)		Number (percent)		Number (percent)
Total	88 (100%)	29 (32.95%)		1 (1.13%)		30 (34.09%)
Age						
10-20	15 (17.04%)	3 (20%)	0.711	0 (0%)	0.775	3 (20%)
21-30	42 (47.72%)	15 (35.71%)		1 (2.38%)		16 (38.09%)
31-40	26 (29.54%)	9 (34.61%)		0 (0%)		9 (34.61%)
41-50	5 (5.68%)	2 (40%)		0 (0%)		2 (40%)
Residence						
Urban	56 (63.63%)	17 (30.35%)	0.493	0 (0%)	0.364	17 (30.35%)
Rural	32 (36.37%)	12 (37.50%)		1 (3.12%)		13 (40.62%)
Education level						
Diploma or lower	59 (67.05%)	22 (37.28%)	0.217	1 (1.69%)	0.481	23 (38.98%)
University degree	29 (32.95%)	7 (24.13%)		0 (0%)		7 (24.13%)
Contact with cat						
Yes	39 (44.31%)	15 (38.46%)	0.327	1 (2.56%)	0.443	16 (41.02%)
No	49 (55.69%)	14 (28.57%)		0 (0%)		14 (28.57%)
Consumption of raw/undercooked meat						
Yes	34 (38.63%)	17 (50%)	0.007	1 (2.94%)	0.386	18 (52.94%)
No	54 (61.37%)	12 (22.22%)		0 (0%)		12 (22.22%)
Source of drinking water						
Purified water	65 (73.87%)	18 (27.69%)	0.077	0 (0%)	0.75	18 (27.69%)
Unpurified water	23 (26.13%)	11 (47.82%)		1 (4.34%)		12 (52.17%)
Reason for referral						
Routine checkup	78 (88.63%)	25 (32.05%)	0.724	0 (0%)	0.898	25 (32.05%)
Abortion	10 (11.36%)	4 (40%)		1 (1.13%)		5 (50%)
History of abortion						
Yes	20 (22.73%)	11 (55%)	0.017	1 (5%)	0.277	12 (60%)
No	68 (77.27%)	18 (26.47%)		0 (0%)		18 (26.47%)

## Data Availability

The data used to support the findings of this study are available from the corresponding author upon reasonable request.

## Abbreviations

ELISA: Enzyme-linked immunosorbent assay
IgG: Immunoglobulin G
IgM: Immunoglobulin M
*T. gondii*: *Toxoplasma gondii.*
